# A Review of Deep Learning Methods for Irregularly Sampled Medical Time Series Data

**DOI:** 10.34133/hds.0456

**Published:** 2026-05-04

**Authors:** Chenxi Sun, Moxian Song, Derun Cai, Baofeng Zhang, Hongyan Li, Shenda Hong

**Affiliations:** ^1^ Harvard Medical School, Boston, MA USA.; ^2^ Beth Israel Deaconess Medical Center, Boston, MA, USA.; ^3^ Key Laboratory of Machine Perception (Ministry of Education), Peking University, Beijing, China.; ^4^ School of Intelligence Science and Technology, Peking University, Beijing, China.; ^5^ National Institute of Health Data Science, Peking University, Beijing, China.; ^6^ Institute of Medical Technology, Health Science Center of Peking University, Beijing, China.

## Abstract

**Importance:** Medical time series constitute the largest data type in electronic health records and are often irregularly sampled in real-world clinical settings. Such irregularly sampled medical time series exhibit uneven time intervals, missing observations, and heterogeneous sampling rates, posing substantial challenges for deep learning models. **Highlights:** In this paper, from an irregularity-aware and data-centric perspective, we categorize existing deep learning methods for irregularly sampled medical time series into missing-data-based and raw-data-based approaches. We analyze their theoretical foundations and practical implications and conduct experiments on benchmark and real-world medical datasets to compare their strengths and limitations. **Conclusion:** Based on these analyses, we provide practical recommendations and discuss open problems and future research directions for modeling irregularly sampled medical time series.

## Introduction

In real-world clinical settings, observations are collected at irregular time points influenced by the type of measurement, the patient’s clinical condition, and the availability of clinical staff. Not all clinical variables are measured regularly; hence, medical time series (MTS) is not ideal but irregular, referred to as irregularly sampled MTS (ISMTS). As shown in Table 1, the ISMTS ratio denotes the proportion of ISMTS among all MTS in the dataset (ISMTS/MTS); the missing rate is computed by mapping the time series onto a regular grid determined by the average sampling rate and calculating the proportion of missing time points. Most electronic health record (EHR) datasets [1] have more than 80% ISMTS.

**Table 1.** Real-world EHR datasets and their irregularities

Category	Name	Institution	Diagnosis number	Patient number	ISMTS ratio (%)	Length (d)	Sampling rate (/d)	Missing rate (%)
Composite	MIMIC- III [29]	MIT	651,047	46,520	98.3	0-121	0–480	85.02
	eICU [31]	MIT	626,858	200,859	97.1	0–95	0–480	84.98
	PIC [32]	ZJU	25,378	12,881	95.4	0.9–37.8	0–480	86.02
Specific	HiRID [132]	ETH	Circulatory	55,602	100	0–84	0–720	81.43
	CINC- 2012 [89]	PhysioNet	Mortality	4,000	100	2	0.5–6.3	80.67
	CINC- 2019 [33]	PhysioNet	Sepsis	30,336	100	0.3–24	0–24	23.42
	COVID- 19 [34]	HUST	COVID-19	485	100	0.1–35.2	0–6	85.00

ISMTS, irregularly sampled medical time series case; MIMIC-III, medical information mart for intensive care III; eICU, electronic intensive care unit; PIC, pediatric intensive care; HiRID, high-time-resolution ICU dataset; CinC-2012, computing in cardiology challenge 2012; CinC-2019, computing in cardiology challenge 2019; MIT, Massachusetts Institute of Technology; ZJU, Zhejiang University; ETH, Swiss Federal Institute of Technology; PhysioNet, physiologic signal repository.

**Fig. 1.**
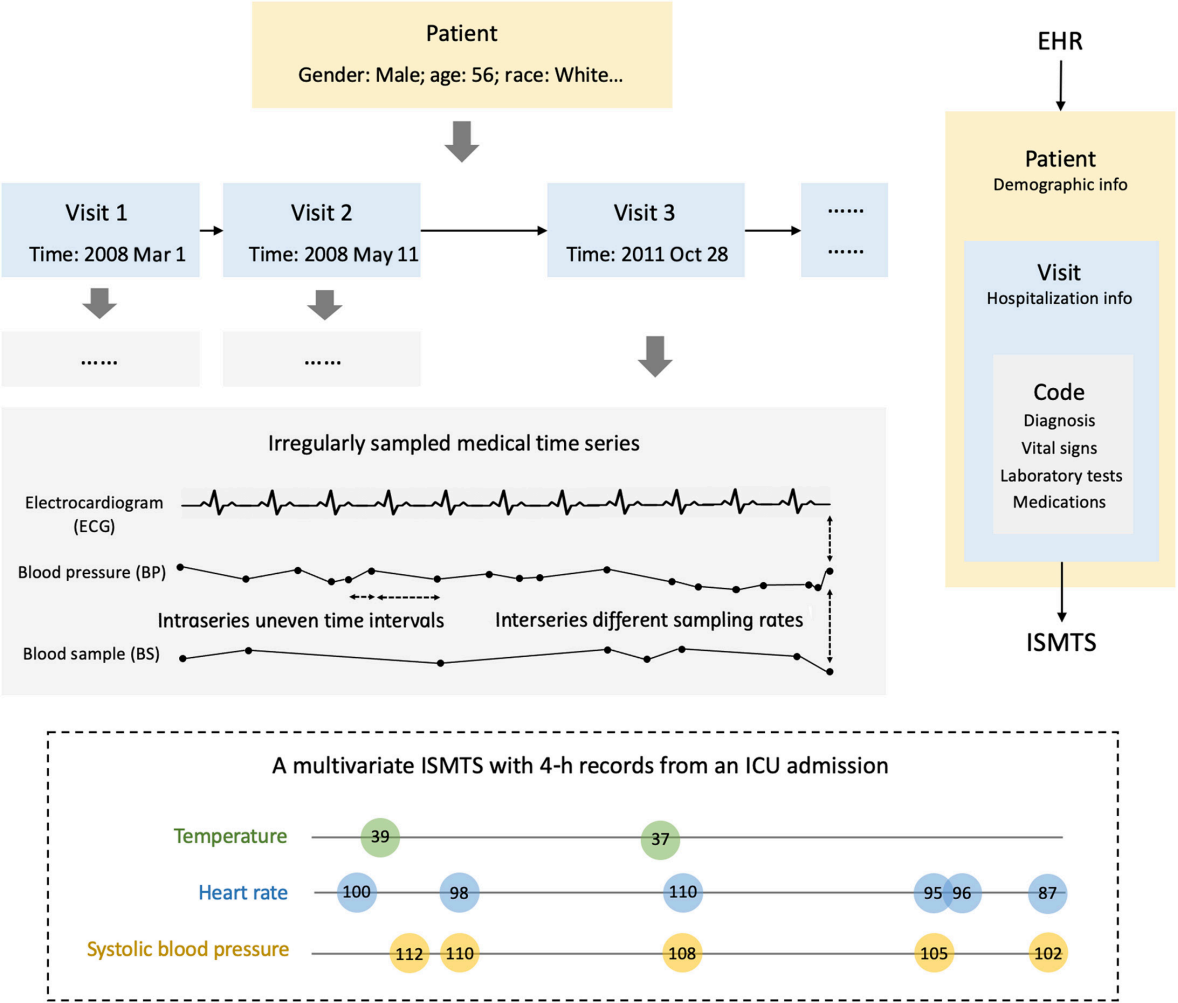
An irregularly sampled medical time series case (ISMTS) in medical information mart for intensive care III (MIMIC-III) electronic health record.

Different from the even MTS, ISMTS has 2 kinds of irregularities as illustrated in Fig. 1:

• Intraseries uneven time intervals (sporadicity and missingness). In a single sequence of ISMTS, time intervals between 2 adjacent observations are different. Medical measurement requirements are usually changed in time depending on the underlying condition of the patient, and missing values exist because of the broken monitor or damaged storage. For example, time intervals between 2 adjacent measurements of blood pressure could be several hours or even several days with the disease progression.
• Interseries multiple sampling rates (variousness and sparsity). Among different sequences of ISMTS, the sampling rates are different. Different medical signals have distinct rhythms and come from sources with inconstant sampling rates. When the frequently sampled vital signs and rarely detected indicators are considered on the same time scale, the latter becomes sparse. For example, the electrocardiogram is collected in seconds, while blood samples are taken in days.

Nowadays, deep learning (DL) [2] technologies have improved healthcare services in automatic diagnosis [2–8], treatment [7,9–11], and scheduling [12–14]. Their success benefits from the rich and available EHRs, which meet the need for large sets of data when training the model. However, most methods assume that MTS is even and complete. In ISMTS, observations differ in number and are not temporally aligned, making it challenging to apply DL models designed for fixed-dimensional feature spaces. In detail, when modeling ISMTS, DL models always ignore at least one of the above 2 irregularities, introduce redundant dependency while suppressing fine-grained information, add undesirable noise or overhead, and ultimately fail in medical tasks.

In this paper, we systematically analyze research from 2015 to 2025 May, focusing on how existing work addresses these challenges along 3 major directions.

• Pondering over the origin of data irregularity. At present, there are 2 understandings about ISMTS’s characteristics. We divide them into 2 categories: missing-data-based methods and raw-data-based methods (this distinction reflects a core modeling assumption rather than a specific architecture or task). The first treats the uneven time interval as having missing observations [15–19]. They solve the problem through more accurate data imputation and interpolation, which can alleviate data sparsity and calculate important data points and sequence rules. The second focuses on the structure of raw data itself [20–23]. They process ISMTS directly through utilizing the uneven time information and handling nonuniform sampling rates, where the sequence dependence is maintained without artificially introducing new relations. Neither view can defeat the other.
• Taking tasks as the hitting point. Two main medical tasks for ISMTS are data imputation (sign control, dose advice, representation, etc.) and outcome prediction (diagnosis, prognosis, recommendation, etc.). The first aims to fill in the missing data, completing ISMTS with more values [24–27]; the second is the downstream medical task with final goals, directly giving the outcome through the input ISMTS [28]. Actually, these 2 kinds of methods can be intertwined. By switching the end-to-end prediction to a 2-stage process, data imputation can be used as a precursor to downstream tasks. In addition, downstream tasks can be additional losses, multiobjective, or assessment tools to make the imputation more logical.
• Fitting for a specific dataset or specific task. The performance of a DL model could vary greatly across datasets. There are many comprehensive EHR datasets such as medical information mart for intensive care III (MIMIC-III) [29] (MIMIC-IV [30], new version), electronic intensive care unit (eICU) [31], and pediatric intensive care (PIC) [32]. There are also many EHR datasets dedicated to specific diseases, such as sepsis [33], COVID-19 [34], and cardiology [35]. Many DL methods are designed for a single disease, with comprehensive datasets serving as supplements [36–39]. Although these specifically targeted scenarios are currently more practical and effective, the general artificial intelligence model seems to be the future direction, but present research is insufficient to support the implementation of a universal medical intelligence model.

The precise procedure depends on the circumstances. Achieving a compromise approach is challenging. For example, sometimes, the data imputation must be introduced when the ISMTS is too irregular to carry out the end-to-end prediction; sometimes, adding the data imputation before the downstream task could result in additional computation and redundant information.

Although various DL approaches for processing ISMTS data have been developed, there is still no systematic review that comprehensively evaluates, summarizes, and guides the field. Moreover, existing surveys do not specifically focus on DL methods or on the analysis of irregular sampling in MTS.

In what follows, through technological reviews and comparative experiments, we categorize related work, highlight their characteristics, summarize challenges and opportunities, and give recommendations:

• The 2 kinds of methods are applicable to different settings. Raw-data-based methods respect the original dependency of ISMTS and produce more accurate experimental results in most circumstances; missing-data-based methods promote data alignment and are better suited for multivariate ISMTS with small sampling rate disparities.
• The additional task can help achieve the target task. Outcome prediction can improve the precision of data imputation for ISMTS. DL models will achieve better ISMTS processing effect in balancing the 2 tasks.
• Different DL structures behave differently. Recurrent neural networks (RNNs) and convolutional neural networks (CNNs) are more stable and practical, whereas self-attention networks (SANs or Transformer) are more potential and currently popular. All methods should be capable of dealing with multivariate MTS and account for both 2 irregularities.
• A better framework should have properties of explainability, reliability, and generality in medical scenarios. In some cases, based on ISMTS, it can be realized through DL’s interpretability method, results’ uncertainty measurement, and tasks’ transference.

## ISMTS in EHRs

**Definition 1**. *EHRs*. An EHR dataset consists of a hierarchical patient–visit–code structure P−V−C, shown in Fig. 1. The basic composition is patient records P. A patient P∈P has its demographic information I∈I and some visits/admissions V⊆V. A visit *V* has many codes C⊆C, including static items *S* (diagnosis, medications, and etc.) and time series *X* (vital signs, laboratory tests, and etc.).

**Definition 2**. *ISMTS*. An multivariate ISMTS $X={\left\{x^{d}\right\}}_{d=1}^{D}$ has *D* dimensions and a label *Y*. Each dimension is a time series $x^{d}={\left\{x_{i}^{d}\right\}}_{i=1}^{N}$ with *N* observations/medical measurements. In addition, each observation $x_{i}^{d}$ corresponds to a time stamp $t_{i}^{d}$. ISMTS has 2 irregularities: (a) uneven time intervals in intraseries, $\Delta_{i}\neq \Delta_{j},\Delta_{i}=t_{i}-t_{i-1},2\leq i,j<T,i\neq j$, and (b) different sampling rates in interseries, $r_{s}\left(x^{i}\right)\neq r_{s}\left(x^{j}\right),r_{s}\left(x^{i}\right)=\frac{|x^{i}|}{T}$. In the data missing perspective, after aligning dimensions and time stamps, $X={\left\{x_{i}^{d}\right\}}_{i,d=1}^{N,D}$ has a mask $M={\left\{m_{i}^{d}\right\}}_{i,d=1}^{N,D}$, recording the absence 0 or presence 1, $m_{i}^{d}\in \left\{0,1\right\}$.

Some openly available, real-world, comprehensive EHR datasets and specific disease datasets, which contain ISMTS data, are summarized in Table 1. An EHR has many ISMTS data based on Definitions 1 and 2. This is due to the complexity of the underlying observation processes that can include normal variation in observation times combined with extended, block-structured periods of missingness. As shown in Fig. 1, ISMTS exists at EHRs’ 2 levels:

At the visit level, a patient will visit hospitals at varying time intervals because of the dynamics of health status. Intervals between 2 visits could be 1 month or even 5 years.

At the code level, the measurement requirements are changing on the basis of the symptom severity during a hospitalization. The intervals between 2 adjacent blood pressure collections could be 1 h or even 7 d, electrocardiogram has a sampling rate in seconds, while blood sample is measured in days.

An ISMTS is usually a multivariate time series, where each variate/dimension is a single MTS. As shown in Fig. 1, a triple-variate ISMTS has 3 MTS of temperature, heart rate, and systolic blood pressure, which is sporadically collected in various healthcare settings where different measurements are recorded for patients throughout their course of stay. A patient’s condition may demand observing only a subset of variables of interest. In addition, the observed time-series matrices are very sparse, as some variables may be measured more frequently than others for a given patient. Not all the variables are observed for every patient when aligning such multivariate time series. The time intervals add a time sparsity factor when they are large. Thus, the ISMTS with multivariate time series has pronounced irregular characteristics in both intra- and interseries.

## Categorization based on Model Architecture

Recent studies have positioned DL as a primary approach for time-series modeling, consistently demonstrating superior performance compared to traditional statistical and classical machine learning methods [40]. Table 2 organizes DL models related to MTS processing based on neural network architecture.

**Table 2.** Model summary for modeling ISMTS data

Network type	Model structure	Downstream prediction task	Imputation task for missing data
RNNs	–	[10,15,17,21–23,45,79,87,106,133–139]	[15,18,19,22,79,80,87,140]
	Bidirection	[23,81,110]	[81,110]
	Attention	[20,107,141,142]	–
	AE	[23,99,107,108,142,143]	[99,107,144,145]
	GAN	[16,25,146]	[25–27,84,85,145,147]
CNNs	–	[21,134,138,148]	–
	AE	[105]	–
	GAN	–	[27]
SANs	Transformer/attention	[51,52,55,63,95,109,111–114,149,150]	[109]
DBNs	–	[87,88]	[87]
GNNs	–	[55–57,62]	–

RNNs, recurrent neural networks; AE, autoencoder; GAN, generative adversarial network; CNNs, convolutional neural networks; SANs, self-attention networks; DBNs, deep belief networks; GNNs, graph neural networks.

### Recurrent neural networks

RNNs are good at learning the information in sequential order so that they usually perform well in modeling time series. A standard RNN processes sequential data by iteratively updating its hidden state along the temporal dimension, with units connected through recurrent transitions. Gated RNN variants are designed to preserve internal memory via feedback mechanisms, making them well suited for modeling temporal dependencies and accommodating variable-length inputs. Long short-term memory (LSTM) [41] and gated recurrent units (GRUs) [42] enhance RNN architectures by enabling more effective modeling of long-term dependencies, contributing to the widespread use of RNN-based methods in MTS analysis. However, standard RNNs are inherently suited for regularly sampled data. In practice, ISMTS are often transformed into regularly spaced sequences through temporal discretization, generating fixed-dimensional inputs for RNNs. Such transformations depend on heuristic design choices, including windowing and interpolation, which may degrade data quality by introducing noise or discarding information.

### Convolutional neural networks

CNNs leverage layered convolutional filters to extract hierarchical and multiscale features. When applied to time series, one-dimensional CNNs operate along the temporal axis using sliding kernels to learn sequential structures in either the time or frequency domain. As an example, temporal convolutional networks (TCNs) [43] capture long-range temporal relationships through stacked temporal convolutions. WaveNet [44] is adapted for modeling long-term dependency and multiple dimensions of multivariate time series by stacked dilated convolutions. Besides, to overcome the limitation of discrete, some methods (e.g., SplineNet [45]) extend convolution into a continuous domain by representing ISMTS as piecewise spline functions and performing continuous convolution or shape-matching directly on these curves. These approaches avoid the need for resampling, preserve the original temporal geometry, and provide a more flexible alternative to CNNs when dealing with irregular MTS. In practice, CNNs focus on learning localized temporal patterns in MTS by applying fixed-size kernels to short segments of EHR data, capturing motifs such as co-occurring conditions and patient similarity. This localized receptive field makes it difficult to effectively model global temporal dependencies.

### Self-attention networks

SANs, particularly Transformer models [46], have achieved remarkable success in sequential modeling tasks. Since Transformers lack inherent knowledge of sequence order, positional encoding is typically introduced to incorporate temporal information. To adapt Transformers for time-series analysis, various extensions have been proposed. For example, Li et al. [47] address challenges in long time series using convolutional self-attention, while Ma et al. [48] use cross-attention across multiple dimensions for multivariate time series. Song et al. [49] integrate multihead self-attention with masking mechanisms to handle missing values. For ISMTS, positional encoding is often replaced by time encoding, combined with feature embeddings and missing indicators [50]. More recently, TEE4EHR [51] learns representations for irregular EHR data by integrating a Transformer-based event encoder (TEE) with a neural point-process objective and attention mechanisms for continuous measurements. Despite these advances, Transformers face limitations in modeling time series. In particular, they lack recurrent inductive biases, making it difficult to capture temporal dynamics effectively, and embedding precise time-step information remains challenging. Although self-attention enables fully parallel computation and offers efficiency advantages over RNNs, it incurs quadratic time and memory complexity with respect to sequence length. This makes modeling very long sequences (e.g., thousands of time steps) computationally demanding [40]. Another emerging direction is to transform time series into image-like representations. For instance,time series as images (TS2I) [52] converts irregular multivariate signals into grids of line-plot subfigures, enabling the use of pretrained vision models such as Swin Transformer and Vision Transformer for downstream classification tasks.

### Deep belief networks

Deep belief networks (DBNs), composed of hierarchical restricted Boltzmann machines (RBMs) [53], are capable of modeling the probability distribution of input data. Their contrastive divergence training procedure enables relatively efficient learning compared to other ISMTS models. However, DBNs are not naturally suited for time-series modeling and are often combined with other architectures. Extensions such as temporal RBMs and RNN-RBM hybrids have been proposed as mainstream approaches for sequential data modeling [54].

### Graph neural networks

Graph neural networks (GNNs) model dependencies in graph-structured data through message passing between nodes. When applying GNNs to model time series, the graph can be built to model the relationship of each observation, to represent the relationship among different sequences in multivariate time series [55], or to fuse different information of value, time, and missingness [56]. Graph Spatiotemporal Process (GST-Pro) [57] models irregular multivariate time series as continuous paths using a graph spatiotemporal neural controlled differential equation and detects anomalies with a parameter-free, distribution-based scorer that evaluates only the model’s forecasts.

### Combined models

Combined models can integrate the strengths and special properties of different DL models. For example, RNNs can model the order information and sequence dependency, and CNNs can learn multiple representative local features. Some hybrid architectures such as RESTFul [58] and convLSTM [59] use RNN to encode temporal patterns of each sequence and then use CNN to model interdependencies between sequences with different time resolutions. Multivariate long short-term memory fully convolutional network (MLSTM-FCN) [60] also integrates the attention mechanism. Temporal graph ordinary differential equation (ODE) [61] introduces a continuous-time message-passing framework that models temporal graphs with irregular sampling by learning an ODE over node states, enabling forecasting without requiring interpolation. WaveGNN [62] uses a Transformer for each sensor’s time series and a dynamic GNN to model relations between sensors, enabling forecasting on irregular multivariate time series without interpolation. Hybrid element-wise Transformer vector-quantized variational autoencoder (HET-VQVAE) [63] uses a HET module that models sparse values, missingness, and temporal irregularity, and a VQVAE discretizes latent states to achieve stable and robust representations for clinical prediction tasks.

### Foundation models

In recent years, many methods have started following the foundation-model paradigm, shifting from task-specific architectures to pretraining on ISMTS. Rather than designing new encoders, these approaches focus on learning generalizable representations through large-scale self-supervised objectives. Examples include LSTM-based frameworks that automatically search for effective pretext tasks (e.g., pretraining and augmentation for ISMTS [64]) and Transformer-based pretraining models (e.g., PrimeNet [65] and pretrained language models for irregularly sampled time series (ISTS-PLM) [66]). This trend highlights a growing view that irregular time series could benefit substantially from representation learning and large-model pretraining across diverse downstream tasks.

## Categorization based on Irregularity Perspective

**Fig. 2.**
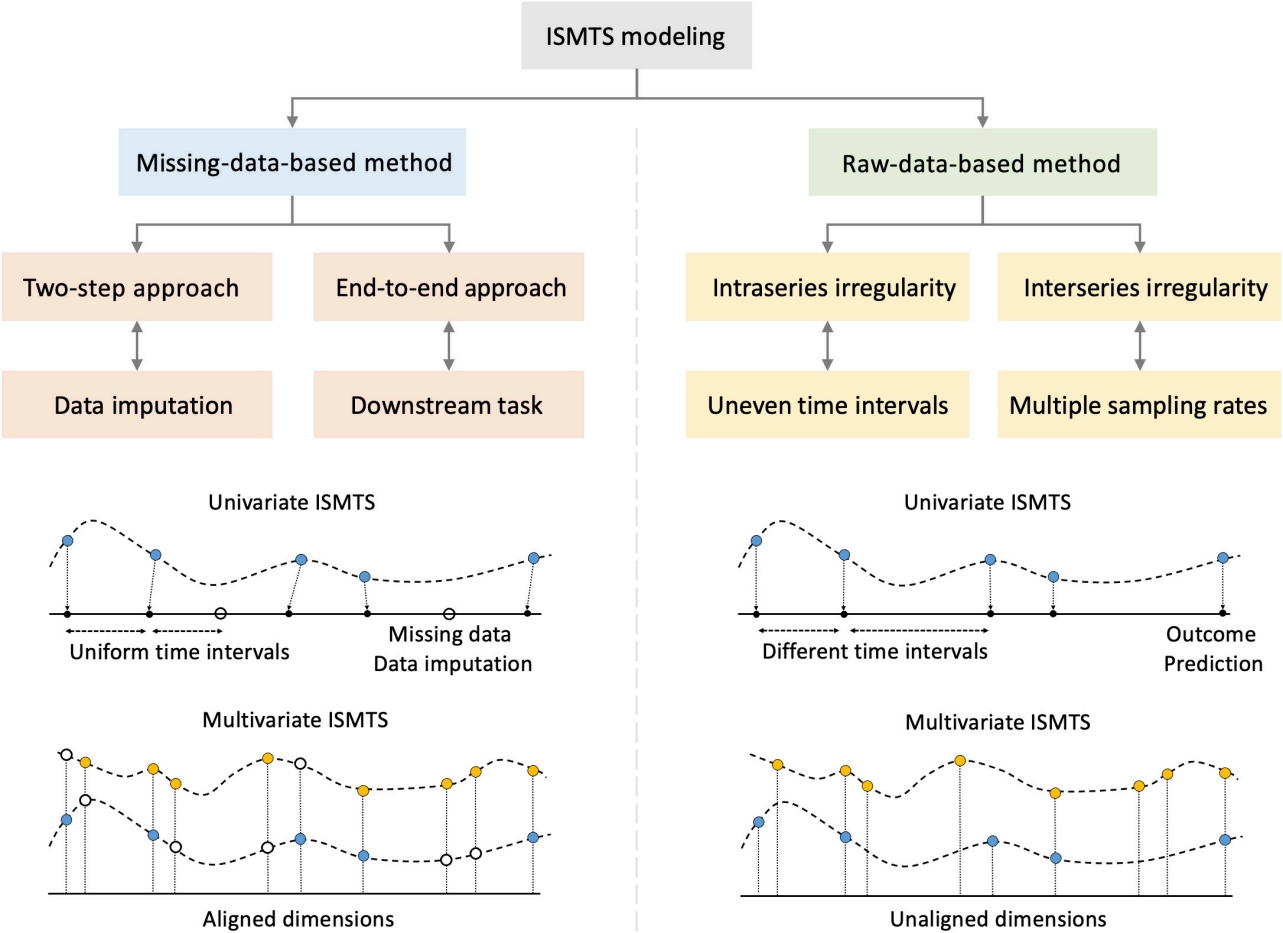
Categorization of related work.

We divide the related work into 2 categories based on their perspective on the source of data irregularity as shown in Fig. 2, with a summary of their respective advantages and disadvantages provided in Table 3. According to one sort of method, irregular features result from missing data, whereas according to the other, they originate from the data itself.

**Table 3.** Summary of advantages and disadvantages of relevant work

Category	Characteristic	Content	
Missing-data-based methods	Two-step	Paper	[18,19,25–27,70,84,85,87,92,147,151,152]
		Advantage	Generality, ability of imputation
		Disadvantage	Suboptimal prediction, incomplete data relation, data generation patterns assumptions, introduced artificial dependency
	End-to-end	Paper	[15–17,45,95,109,110,133]
		Advantage	Optimal prediction
		Disadvantage	Noncommonality, introduced artificial dependency
Raw-data-based methods	Intraseries	Paper	[10,20,21,52,55,61,63,108,113,139,142,143,149,153–156]
		Advantage	No artificial dependency
		Disadvantage	Low-applicability for multivariate data, incomplete data relation
	Interseries	Paper	[22,23,88,99,100,107,111,113,114,138,141,150]
		Advantage	No artificial dependency, relatively complete relations
		Disadvantage	Complex implementation, data generation patterns assumptions

### Missing-data-based methods

These methods regard that ISMTS has a uniform time interval. As shown in Fig. 2, the time axis is segmented into disjoint intervals without overlap. The time points without observed values are considered to be the missing data points [67–69]. The missing rate $r_{m}=\frac{|M_{m}|}{|M|},M_{m}=\left\{m_{i}|m_{i}=0\right\}$ can measure the missing degree at a given sampling rate $r_{s}=\frac{|M_{s}|}{T},M_{s}=\left\{m_{i}|m_{i}=1\right\}$. In this perspective, real-world EHRs have a serious missing data issue. For example, the global missing rates for MIMIC-III, computing in cardiology challenge 2012 (CINC-2012), CINC-2019, and COVID-19 datasets are 85.02%, 80.67%, 23.42%, and 85.00%, respectively.

Data missing problem harms the temporal dependencies of sequences and renders many existing DL models directly inapplicable [25]. To solve this problem, many prior efforts have been dedicated. Here, we divide them into 2 categories: 2-step approaches and end-to-end approaches.

#### Two-step approaches

Two-step approaches have 2 separate processing steps. The first step is to impute the missing values in ISMTS, and the second step is to achieve the medical task based on that preprocessed data. The first step is more important because it determines the quality of the data used to train models [70,71]. As DL models have become active, getting large amounts of complete data has become an important issue [26].

Classical data-filling methods [72–77] cannot capture the correlation between variables and complex patterns, and simple reasoning design with necessary model assumptions results in inaccurate data imputation. Deep neural networks perform better. For example, RNNs can impute data by modeling recursive relations in the direction of sequence evolution [78]. In addition, the effectiveness of LSTM and GRU has been demonstrated [19,79,80]. However, most of them learn an incomplete relation by only considering intraseries or interseries relations. Multidirectional RNN [81] first noticed the difference between these 2 relations, used bidirectional RNN [82] to interpolate values in the intraseries direction and used multilayer perceptron to impute values in the interseries direction. However, the estimated values are treated as constants that cannot be sufficiently updated, and the relation between missing variables, which also contains rich features, is dropped [18].

Besides, missing values can be generated in addition to being imputed. Generative adversarial networks (GANs) [83] can generate complete time series without missing data through an adversarial process of the generator and discriminator. Generative adversarial imputation nets (GAINs) [84] receive the mask *M* as input to present missing data. The ensemble of multiple pairs of generator–discriminator and that of multi-inputs/outputs can strengthen GAIN [85,86]. Furthermore, Bayesian inference provides a natural framework for continual learning by updating the posterior distribution of model parameters, as new time-series data are observed. Consequently, DBNs can be extended and adapted for modeling ISMTS [87,88].

The main goal of the 2-step method is to estimate the missing values. However, in the medical background, the ultimate goal is to carry out final tasks such as medical prediction [89,90], concept representation [6], and patient subtyping [7,10,11]. Two separate steps may result in suboptimal analyses and predictions caused by the not effectively explored missing patterns due to the separation of imputations and final tasks [91].

#### End-to-end approaches

End-to-end approaches directly process medical tasks without filling in the missing values of ISMTS. Their goal is to predict, classify, or cluster data, whereas data imputation is an additional task or even not a task at all. For example, Dated GRU [15] is equipped with $X^{*}\leftarrow \left[X,\;\Delta ,\;M\right]$ as the indicator for missing data. Meanwhile, the missing rate is always correlated with the labels, demonstrating the utility of missingness patterns in solving the final task $Y\leftarrow \left[X,\;X^{*}\right]$ [21,28]. In addition, bidirectional RNNs can represent both forward and backward value couplings within a time series, thus modeling more relations [23,81].

However, they are limited to using local information, such as assuming that a missing variable can be represented as a combination of the last observed value and the mean value. The local statistics are unreliable when the continuous data misses or the missing rate rises up. The absence of continuous data and important signals adversely affects data quality, resulting in unstable predictions and other unpredictable effects [92]. For example, the signal valley that has never been observed before cannot be inferred by methods that only model local relations. However, the valley values of blood pressure are important for patients to indicate sepsis, a leading cause of patient mortality in intensive care unit [93]. Global and local network [16] solves this problem using a memory module for global ISMTS structure.

From a missing-data perspective, ISMTS is typically aggregated into discrete time intervals and then imputed with estimated values. However, this process may obscure fine-grained temporal information, as the resolution of observed MTS varies across patients depending on their underlying conditions. In addition, such transformations can introduce artificial temporal dependencies, inject noise, and increase computational overhead. These issues are exacerbated as the data become sparser, ultimately leading DL models to learn representations that may not align with the target task.

### Raw-data-based methods

These methods pay more attention to the characteristics of the data itself by directly receiving ISMTS as input. They primarily model intraseries and interseries relations.

#### Intraseries-irregularity-based approaches

Intraseries-irregularity-based approaches aim to model uneven time intervals, which are prevalent in real-world EHR data. For example, even relatively clean datasets such as the sepsis dataset contain more than 60% of time series with irregular sampling. As illustrated in Fig. 2, these approaches do not impute missing values to enforce regularity. Instead, they treat temporal irregularity itself as informative and explicitly encode it in the model.

However, there is a hypothesis of DL that the time differences in the sequences are distributed evenly and model the uneven time interval [41,94], e.g., modern RNNs typically assume regular sampling rates. To address this, Baytas et al. [10] propose time-aware LSTM to change the sequential state in classical LSTM by implementing a memory discount with the time decay function $g\left(\Delta \right)=\frac{1}{\text{log}\left(e+\Delta \right)}$. Self-attention-based methods [95] jointly model the relationships among observed values, missing indicators, and the time intervals between consecutive observations. Beyond modifications to RNN gating mechanisms, recent work has explored neural ODEs [96], which model the evolution of hidden states in continuous time between observations, while updating them using standard gating structures [97,98]. However, ODE-based approaches often incur substantial computational overhead due to the need for numerical integration. To address this limitation, Schirmer et al. [99] proposed the continuous recurrent unit, which leverages stochastic differential equations and Kalman filtering to avoid expensive numerical integration and variational approximations. Neural continuous-discrete state space model [100] uses stochastic differential equations to model latent dynamics and a linear-Gaussian structure to incorporate discrete observations. ProFITi [63] is a conditional normalizing flow model that estimates the full joint distribution of multivariate time series with irregular sampling and missing values, using an invertible triangular attention mechanism and a novel invertible activation function.

However, modeling multivariate ISMTS often requires aligning multiple time series and performing missing value imputation as a preprocessing step. Such approaches typically assign equal importance to observed and imputed data, disregarding the lower reliability of imputed values. To mitigate this limitation, dual-attention time-aware GRU [20] proposes an unreliability-aware attention mechanism that incorporates both data quality and domain knowledge. In addition, attention-based models improve interpretability, a critical requirement in medical applications. Since clinical events in EHR data may be concentrated within a single admission or spread across multiple admissions, data augmentation methods such as temporal coarsening [21] have been introduced to exploit temporal clustering invariance, thereby improving model robustness for multivariate MTS.

#### Interseries-irregularity-based approaches

Interseries-irregularity-based approaches aim to model multiple sampling rates, which are also common in the real-world EHRs. For example, in the MIMIC-III dataset, only 1.68% of the samples have the same average sampling rate as others. ISMTS data exhibit dependencies both within single time series and across different variables. These cross-series interactions are often informative and essential for downstream analysis. For example, a patient’s blood pressure at a given time depends not only on its own temporal trajectory but also on its relationship with other signals, such as heart rate, across time.

Although some techniques have integrated the global structures, they either process time series with a stable sampling rate [101,102] or incorporate the information from all time points into an interpolation model [103,104], which is redundant and low adaptive with the complicated and expensive computation. Multirate hierarchical deep Markov model and autoregressive framework [23,105] are proposed to learn multirate multivariate MTS. Horn et al. [50] consider the interseries irregularity to be caused by unaligned measurements. They implement differentiable set function learning to align multivariate time series. However, they just considered the different sampling rates between series but ignored the uneven time intervals within the series.

A multivariate ISMTS can be treated as multiple MTS sequences with different sampling rate. It is feasible to process a single MTS first and then integrate. For example, interpolation-prediction network [22] uses this idea to make up for the lack of modularity and address the difficulty of the interpolation complexity. Time-encoding echo state network [106] proposes the time decay function to encode intraseries irregularity and the skip connection to fuse interseries irregularity. Besides, the attention mechanism attends only to the observed data dimensions at each time point and hence does not require a separate imputation step to handle vector-valued observations with an arbitrary collection of dimensions missing at any given time point. Shukla et al. [107] introduce the multitime attention network (mTAN), a variational autoencoder (VAE) architecture for continuous-time modeling. In addition, initial value problem VAE (IVP-VAE) [108] improves the efficiency of latent ODE/flow models by replacing sequential ODE evolution with parallel IVP solving and reusing an invertible IVP solver for both the encoder and decoder.

Similar to many VAE-based frameworks, mTAN assumes a homoscedastic output distribution conditioned on latent variables, which limits its ability to directly model input-dependent uncertainty. As a consequence, variability due to sparse observations is only implicitly reflected in the latent space. To address this issue, heteroscedastic extensions have been proposed to explicitly capture uncertainty that varies with the input [109]. In addition, a growing body of work focuses on modeling cross-variable dependencies in multivariate ISMTS. Bidirectional coupled MTS learning [110] captures bidirectional interactions across variables using self-attention. Dual-attention and memory-augmented networks [111] augments intraseries representations with an interseries attention module to model dependencies across variables and time. Warpformer [112] introduces learnable temporal warping combined with dual self-attention to capture both multiscale temporal patterns and cross-variable relationships. Compatible Transformer [113] represents each observation as a variate-time token and alternates between intravariate and intervariate attention layers, enabling joint modeling of temporal dynamics and variable interactions without relying on interpolation. MuSiCNet [114] converts ISMTS into hierarchical scales and learns representations using cross-scale rectification.

## Empirical Experiments

We further implement some representative methods and carry out experiments to compare their performances. Experiments are based on 4 real-world datasets for imputation task and classification task.

All baseline methods were implemented using publicly available code. To ensure a fair and reproducible comparison, each model was retrained using the hyperparameter settings reported in the corresponding original publications. Methods without publicly available implementations or clearly documented hyperparameters were not included in the experimental comparison.

The experiments are based on 2 comprehensive datasets—MIMIC-III [29] and CINC-2012 [89]—and 2 specific datasets—CINC-2019 [33] and COVID-19 [34]. The tasks are medical data imputation (estimating missing values by randomly masking observations at the time-point level), disease classification, mortality prediction, sepsis diagnosis before 6 h, and COVID-19 mortality prediction. The imputation results are evaluated by root mean square error (RMSE) in Eq. (1). Model performance for classification tasks is assessed using receiver operating characteristic-area under curve [Eq. (2)], which summarizes the trade-off between the true-positive rate (TPR) and false-positive rate (FPR). The quantities TP, TN, FP, and FN denote true positives, true negatives, false positives, and false negatives, respectively.

$$\text{RMSE}=\sqrt{\frac{1}{N_{\text{missing}}}\sum_{i=1}^{N_{\text{missing}}}{\left(x_{i}-{\hat{x}}_{i}\right)}^{2}}$$ (1)

$$\mathrm{TPR}=\frac{\mathrm{TP}}{\mathrm{TP}+\mathrm{FN}},\;\mathrm{FPR}=\frac{\mathrm{FP}}{\mathrm{TN}+\mathrm{FP}}$$ (2)

### Medical data imputation task

The results of baselines in imputation tasks are shown in Table 4 (the smaller the value of RMSE, the better). There is little difference between RNN-based and GAN-based methods in terms of accuracy. They all perform well on the sepsis and COVID-19 datasets. Perhaps, compared to the other 2 datasets, the time series in these 2 datasets are from patients with the same disease, and the data are relatively clean and evenly distributed.

**Table 4.** Method performances in medical data imputation task (RMSE↓, underlined values indicate the best performance, and bold values indicate the second-best.). The imputation task is to impute values in time points, which have no observed value after discretizing time axis into nonoverlapping intervals.

		MIMIC-III	CINC-2012	CINC-2019	COVID-19
RNNs	RNN [17]	4.985 ± 0.118	4.180 ± 0.118	2.901 ± 0.062	1.685 ± 0.038
	LSTM [19]	4.712 ± 0.120	4.046 ± 0.123	2.899 ± 0.091	1.710 ± 0.035
	LIME-RNN [157]	4.210 ± 0.123	3.546 ± 0.130	2.363 ± 0.082	1.549 ± 0.071
	GRU-D [15]	4.412 ± 0.109	3.567 ± 0.132	2.379 ± 0.084	1.543 ± 0.072
	M-RNN [81]	4.435 ± 0.201	3.236 ± 0.098	2.337 ± 0.067	**1.530 ± 0.056**
	BRITS [18]	4.339 ± 0.187	3.238 ± 0.115	2.439 ± 0.073	1.539 ± 0.034
	TBM [140]	4.542 ± 0.135	3.669 ± 0.162	2.499 ± 0.037	1.603 ± 0.066
	AANN [80]	4.124±0.107	3.360 ± 0.149	2.297 ± 0.030	1.593 ± 0.060
	MR-HDMM [23]	4.015 ± 0.168	3.113 ± 0.100	**2.217 ± 0.095**	1.555 ± 0.073
	B-CIPIT [87]	4.042 ± 0.135	3.250 ± 0.112	2.240 ± 0.030	1.541 ± 0.076
	BiCMTS [110]	4.101 ± 0.117	3.340 ± 0.109	2.231 ± 0.072	1.545 ± 0.029
GANs	GAN [26]	4.342 ± 0.132	4.001 ± 0.120	2.497 ± 0.060	1.703 ± 0.075
	GAIN [84]	4.040 ± 0.130	3.230 ± 0.102	2.225 ± 0.071	1.533 ± 0.061
	GAN-GRUI [25]	3.920 ± 0.156	3.129 ± 0.112	2.225 ± 0.067	1.534 ± 0.052
	MisGAN [27]	4.998 ± 0.133	3.150 ± 0.110	2.249 ± 0.039	1.542 ± 0.048
	Stackelberg [85]	4.431 ± 0.169	3.741 ± 0.097	2.435 ± 0.078	1.932 ± 0.039
	E^2^-GAN [145]	**3.891 ± 0.106**	3.114 ± 0.123	2.208 ± 0.061	1.555 ± 0.066
AE	mTAN [107]	4.140 ± 0.078	3.200 ± 0.101	2.220 ± 0.072	1.556 ± 0.060
	DAE [144]	4.010 ± 0.053	**3.109 ± 0.123**	2.242 ± 0.069	1.601 ± 0.023
	CRU [99]	3.933 ± 0.052	3.114 ± 0.164	2.218 ± 0.067	1.559 ± 0.042
	IVP-VAE [108]	3.911 ± 0.049	3.201 ± 0.101	2.217 ± 0.079	1.548 ± 0.060
Self-attention	Transformer [46]	4.229 ± 0.177	3.825 ± 0.170	2.398 ± 0.143	1.650 ± 0.102
	HeTVAE [109]	4.040 ± 0.143	3.442 ± 0.149	2.718 ± 0.161	1.657 ± 0.101
	MIAM [95]	3.911 ± 0.073	3.201 ± 0.103	2.218 ± 0.075	1.550 ± 0.060

RMSE, root mean square error; RNN, recurrent neural networks; LSTM, long short-term memory; GRU-D, dated gated recurrent unit; M-RNN, multidirectional RNN; BRITS, bidirectional recurrent imputation for time series; MR-HDMM, multirate hierarchical deep Markov model; BiCMTS, bidirectional coupled medical time series learning; GAN, generative adversarial network; GAIN, generative adversarial imputation net; mTAN, multitime attention network; AE, autoencoder; CRU, continuous recurrent unit; IVP-VAE, initial value problem variational autoencoder; MIMIC-III, medical information mart for intensive care III; CinC-2012, computing in cardiology challenge 2012; LIME-RNN, learning individualized models for electronic health records with recurrent neural networks; TBM, temporal belief memory; AANN, adversarial autoencoder neural network; B-CIPIT, bidirectional continuous-time imputation and prediction network; GAN-GRUI, generative adversarial network with gated recurrent unit imputation; MisGAN, missing data generative adversarial network; E2-GAN, end-to-end generative adversarial network; DAE, denoising autoencoder; HeTVAE, heterogeneous time variational autoencoder; MIAM, missingness-informed attention model.

### Medical classification tasks

The results of baselines in outcome prediction tasks are shown in Tables 4 and 5. The methods with 2 stages first use the listed methods to impute missing values and then use the basic model to classify the complete time series; The end-to-end methods directly classify time series through modeling. It is obvious that the latter methods perform better. When modeling and extrapolation are combined with downstream tasks, the results can be superior to separate processes. For end-to-end methods, both types of methods have their own advantages. Basically, raw-data-based methods work relatively better than missing-data-based methods. Meanwhile, methods that take into account both interseries and intraseries relations are more accurate. Besides, the methods show different effects on different datasets. For example, time-aware LSTM performs well on COVID-19, as the relatively simple methods perform better on small datasets.

**Table 5.** Method performances in outcome prediction/classification task (AUC-ROC↑, underlined values indicate the best performance, and bold values indicate the second-best.). The task on CINC-2012 and COVID-19 is mortality prediction; the task on MIMIC-III is 8 disease diagnosis; the task on CINC-2019 is sepsis diagnosis before 6 h.

		MIMIC-III	CINC-2012	CINC-2019	COVID-19
Basic models	RNN [17]	0.809 ± 0.014	0.800 ± 0.016	0.825 ± 0.024	0.943 ± 0.004
	LSTM [19]	0.812 ± 0.009	0.805 ± 0.010	0.829 ± 0.019	0.945 ± 0.005
	1D-CNN [43]	0.802 ± 0.003	0.800 ± 0.012	0.815 ± 0.010	0.931 ± 0.005
	TCN [44]	0.813 ± 0.007	0.807 ± 0.011	0.830 ± 0.021	0.941 ± 0.003
	Transformer [46]	0.814 ± 0.014	0.801 ± 0.012	0.819 ± 0.013	0.932 ± 0.005
	Informer [158]	0.812 ± 0.006	0.804 ± 0.012	0.831 ± 0.015	0.934 ± 0.004
Two stages (imputation + basic model classification)	LIME-RNN [157]	0.814 ± 0.012	0.816 ± 0.010	0.843 ± 0.011	0.942 ± 0.006
	TBM [140]	0.819 ± 0.010	0.813 ± 0.012	0.845 ± 0.020	0.923 ± 0.003
	MR-HDMM [23]	0.829 ± 0.004	0.814 ± 0.005	0.841 ± 0.015	0.925 ± 0.009
	GAIN [84]	0.805 ± 0.005	0.809 ± 0.011	0.822 ± 0.009	0.940 ± 0.005
	GAN-GRUI [25]	0.820 ± 0.005	0.809 ± 0.013	0.830 ± 0.011	0.940 ± 0.006
	MisGAN [27]	0.798 ± 0.012	0.797 ± 0.010	0.809 ± 0.012	0.941 ± 0.007
	Stackelberg [85]	0.798 ± 0.010	0.797 ± 0.010	0.789 ± 0.012	0.941 ± 0.007
	HeTVAE [109]	0.808 ± 0.014	0.817 ± 0.012	0.793 ± 0.011	0.940 ± 0.006
End-to-end (missing-data-based methods)	GRU-D [15]	0.829 ± 0.003	0.824 ± 0.009	0.835 ± 0.013	0.963 ± 0.003
	M-RNN [81]	0.827 ± 0.005	0.820 ± 0.011	0.842 ± 0.010	0.959 ± 0.003
	BRITS [18]	0.832 ± 0.002	0.819 ± 0.012	0.839 ± 0.013	0.959 ± 0.002
	LGnet [16]	0.834 ± 0.003	0.820 ± 0.013	0.843 ± 0.013	0.956 ± 0.002
	BiCMTS [110]	0.834 ± 0.002	0.819 ± 0.013	0.847 ± 0.014	0.960 ± 0.005
	MIAM [95]	0.832 ± 0.005	0.822 ± 0.012	0.849 ± 0.010	0.962 ± 0.003
	STraTS [150]	0.832 ± 0.005	0.819 ± 0.010	0.850 ± 0.013	0.961 ± 0.005
End-to-end (raw-data-based methods)	IPN [22]	0.831 ± 0.003	0.823 ± 0.009	0.844 ± 0.015	0.960 ± 0.003
	T-LSTM [10]	0.817 ± 0.004	0.821 ± 0.010	0.831 ± 0.014	**0.965 ± 0.003**
	DATA-GRU [20]	0.832 ± 0.006	0.823 ± 0.012	0.851 ± 0.012	0.961 ± 0.003
	UA-CRNN [138]	0.830 ± 0.004	0.820 ± 0.015	0.851 ± 0.007	0.956 ± 0.004
	CRU [99]	0.834 ± 0.003	0.821 ± 0.014	0.849 ± 0.011	0.963 ± 0.002
	TE-ESN [106]	0.833 ± 0.004	0.822 ± 0.010	0.850 ± 0.012	**0.965 ± 0.004**
	mTAN [107]	0.834 ± 0.005	0.822 ± 0.012	0.848 ± 0.009	0.960 ± 0.005
	DAMA-Net [111]	0.833 ± 0.002	0.821 ± 0.010	0.850 ± 0.009	0.961 ± 0.004
	NCDSSM [100]	0.836 ± 0.004	0.824 ± 0.011	0.851 ± 0.010	0.963 ± 0.001
	TG-ODE [61]	0.837 ± 0.003	0.825 ± 0.009	**0.852 ± 0.008**	0.963 ± 0.001
	MuSiCNet [114]	0.838 ± 0.004	**0.826** ± **0.010**	**0.852 ± 0.009**	0.963 ± 0.002
	HET-VQVAE [63]	**0.839** ± **0.005**	0.824 ± 0.012	**0.852 ± 0.027**	0.962 ± 0.002
	ViTST [52]	0.835 ± 0.004	0.823 ± 0.011	0.849 ± 0.010	0.961 ± 0.003
	Warpformer [112]	0.836 ± 0.003	0.824 ± 0.010	0.850 ± 0.009	0.964 ± 0.003
	CoFormer [113]	0.830 ± 0.004	0.825 ± 0.012	0.851±0.008	0.962 ± 0.002
	TEE4EHR [51]	0.836 ± 0.005	0.824 ± 0.013	0.850 ± 0.010	0.962 ± 0.003
	ISTS-PLM [66]	0.837 ± 0.004	0.825 ± 0.011	0.851 ± 0.009	0.963 ± 0.002

AUC-ROC, receiver operating characteristic-area under curve; RNN, recurrent neural network; LSTM, long short-term memory; 1D-CNN, one-dimensional convolutional neural network; TCN, temporal convolutional networks; MR-HDMM, multirate hierarchical deep Markov model; GAIN, generative adversarial imputation net; GRU-D, dated gated recurrent unit; M-RNN, multidirectional RNN; BRITS, bidirectional recurrent imputation for time series; LGnet, global and local network; BiCMTS, bidirectional coupled medical time series learning; IPN, interpolation-prediction network; CRU, continuous recurrent unit; TE-ESN, time-encoding echo state network; mTAN, multitime attention network; DAMA-Net, dual-attention and memory-augmented networks; NCDSSM, neural continuous-discrete state space model; TG-ODE, temporal graph ordinary differential equation; HET-VQVAE, hybrid element-wise Transformer vector-quantized variational autoencoder; CoFormer, compatible Transformer; CinC-2012, computing in cardiology challenge 2012; MIMIC-III, medical information mart for intensive care III; LIME-RNN, learning individualized models for electronic health records with recurrent neural networks; TBM, temporal belief memory; GAN-GRUI, generative adversarial network with gated recurrent unit imputation; MisGAN, missing data generative adversarial network; MIAM, missingness-informed attention model; STraTS, sparse time series transformer; T-LSTM, time-aware long short-term memory; DATA-GRU, decay augmented time-aware gated recurrent unit; UA-CRNN, uncertainty-aware convolutional recurrent neural network; ViTST, vision transformer for time series; TEE4EHR, transformer event encoder for electronic health records; ISTS-PLM, irregularly sampled time series pretrained language model.

## Discussion

It is crucial to design efficient DL techniques for modeling the frequently occurring ISMTS in medical contexts. When dealing with this subject, we outlined 4 open problems and provided potential solutions based on the aforementioned studies and experiments.

### Possible responses to open questions

#### How to choose the model? (RNNs, CNNs, SANs, etc.)

The model selection depends on specific application tasks and dataset characteristics. According to the available research and our comparative studies, RNN- and CNN-based models and attention mechanisms perform well in prediction tasks (in-hospital mortality prediction, length of stay prediction, disease diagnosis, phenotype classification, etc.); GAN-based and autoencoder-based network structures perform well in imputation tasks and feature learning tasks (missing value imputation, examination result completion, medical sample generation, etc.); SANs, Transformers, and DBNs perform well in unsupervised or self-unsupervised learning tasks (representation, patient subtyping, medical semantic analysis, etc.); GNNs are more suitable for downstream tasks where the ISMTS is structurally modeled (drug recommendation, dosage control, etc.). Although they all need sufficient training data, RNNs and CNNs are easier to train and converge, while the emerging Transformers are not very stable and demand a larger data volume. However, luckily, different types of DL models can be integrated into the designs of neural networks so as to gather various strengths.

For MTS applications, gated RNN architectures still retain certain advantages compared to more recent models such as Transformers. As discussed earlier, different modeling components are inherently better suited to specific tasks. In particular, unidirectional discrete-time RNNs, due to their filtering characteristics, are more appropriate for detection and prediction tasks rather than interpolation or smoothing. In contrast, bidirectional RNNs combined with attention mechanisms can effectively handle missing value imputation and interpolation by leveraging both past and future context and selectively focusing on relevant parts of the sequence. Another important challenge for RNN-based models is handling incomplete vector-valued inputs. Common strategies include incorporating missing indicators and using time-aware decay functions to account for irregular intervals. Furthermore, methods specifically designed for predictive modeling are less prevalent than those focused on imputation, particularly those based on recurrent GANs and recurrent autoencoders.

#### How to choose the modeling perspective? (missing data or raw data)

Missing-data-based methods are more suitable for medical data interpolation and knowledge discovery due to their properties of fitting and regression; raw-data-based methods have more potential for downstream medical tasks when interpolation is not necessary. Both 2 kinds of methods have advantages and disadvantages. It is difficult to sum up which method is better.

Most existing approaches rely on missing-data formulations, introducing assumptions that may not hold in real-world ISMTS. For example, many methods are evaluated on datasets with relatively low missing rates, limiting their effectiveness in scenarios with high sparsity or multiple missing values. When irregularity is converted into missingness through temporal discretization, the choice of interval length becomes a critical hyperparameter. Larger intervals reduce the number of missing values but aggregate multiple observations within the same interval, whereas smaller intervals increase sparsity and lead to frequent missing entries. Both extremes can degrade model performance, either by obscuring fine-grained information or by introducing excessive sparsity and ambiguity in value selection. Moreover, imputation-based approaches may introduce artificial dependencies, inflate data size due to excessive imputation, and weaken the original temporal relationships through forced multivariate alignment. These limitations motivate the development of irregularity-aware methods that directly operate on sparse and irregularly sampled multivariate time series without relying on imputation. However, raw-data-based approaches often involve complex model designs and large numbers of parameters. Methods focusing on irregular time intervals are generally easier to implement, as they mainly incorporate time-decay mechanisms, but their performance may not consistently meet expectations across different tasks. In addition, in multivariate settings, aligning variables across dimensions can reintroduce missing data issues. Multisampling-rate approaches avoid explicit missingness but typically require more parameters and are less compatible with efficient batch training.

#### How to deal with multiple irregularities? (intraseries and interseries)

For univariate ISMTS, only the intraseries irregular time interval issue needed to be handled using missing-data-based or raw-data-based modeling perspective; for multivariate ISMTS, another interseries multiple sampling rate issue also needs to be handled. If the difference among sampling rates is not large, we can first align multiple variables or dimensions with allowing missing values and then mainly model the intraseries irregularity; if the difference is large, we had better consider multivariate time series as multiple time series, modeling them separately and then fusing them.

The boundaries between these modeling perspectives are not strictly defined. For instance, when treating multivariate ISMTS as a single sample, intraseries relationships can be characterized by both temporal (longitudinal) and cross-variable (horizontal) dependencies. Temporal dependencies capture aspects such as ordering, contextual information, time intervals, and decay dynamics, while horizontal dependencies describe interactions across different variables, where interfeature correlations provide complementary temporal insights. In contrast, interseries relationships emerge from patterns across different samples. When multivariate ISMTS are instead viewed as multiple separate univariate sequences, the nature of these relationships changes. Intraseries dependencies reduce to temporal relationships within each individual sequence, where variations across time intervals must be carefully modeled. Meanwhile, interseries relationships reflect similarities and patterns across different patients or across sequences of the same variable. From a structural perspective, intraseries modeling primarily captures local patterns, whereas interseries modeling focuses on global structures. Jointly modeling both local and global dependencies has been shown to improve performance in tasks such as mortality prediction, although such approaches are typically more complex and may not generalize consistently across datasets.

#### How to balance different tasks? (data imputation and outcome prediction)

For medical prediction tasks, decoupled schemes perform worse than methods that are trained end-to-end. To model ISMTS, many methods utilize the data imputation as an additional task for the downstream task. Two-step approaches first perform data completion and then carry out forecasting. However, such methods often overlook the informative nature of missingness, as the patterns of missing data may themselves contain valuable signals. In particular, evaluating imputation accuracy using randomly generated missing values may fail to reflect real-world missingness mechanisms. In contrast, end-to-end approaches jointly optimize imputation and prediction within a unified framework, allowing the model to leverage data reconstruction in a way that directly benefits the final task.

Further, different modeling approaches tend to be better suited for different tasks. For instance, GAN-based methods are often favored for imputation, whereas RNN-based models are commonly used for prediction. However, such generalizations do not consistently hold across datasets. For example, in the COVID-19 dataset, RNN-based methods outperform GANs in imputing missing values. Similarly, 2-step approaches based on GANs can achieve comparable performance to direct RNN-based methods in mortality prediction tasks. These observations suggest that it is challenging to identify a universally optimal modeling approach for MTS. Instead, model selection should be guided by the specific task requirements and the characteristics of the dataset.

### Directions for future work

#### Data: Transform ISMTS to new data forms

Beyond conventional signal features, such as time- and frequency-domain characteristics, missingness patterns, and sampling irregularities, alternative representations of ISMTS have been explored through signal transformations. In particular, time-series data can be converted into image or graph-based formats. For example, representations such as causality graphs, Markov transition field images, and Gramian angular field images have been applied in electrocardiogram analysis [115]. These transformations open new opportunities for leveraging models from computer vision and natural language processing to analyze MTS data.

#### Model: Develop new DL structures and general models

Most existing methods extend DL models with additional strategies rather than fundamentally redesigning architectures. In recent years, many studies have adapted state-of-the-art models from computer vision and natural language processing, such as ResNet [116] and Informer [117], for time-series modeling. In contrast, relatively few works focus on systematically modifying neural architectures to explicitly handle irregular data, despite their potential as elegant and promising solutions. Nevertheless, emerging approaches—including neural ODEs [40], bistable recurrent cells [118], and spiking neural networks [119]—have demonstrated encouraging results. Leveraging the parallelism and structural properties of these architectures, future work may further explore attention mechanisms and invariance-based designs to improve the trade-off between accuracy and computational efficiency. However, model transferability across diverse datasets remains underexplored. Key characteristics of time series—such as sequence length, volatility, and underlying processes—and properties of missing data—including sparsity, randomness, and interval distribution—require more systematic analysis and quantification. Moreover, most existing DL approaches do not explicitly account for classical missing data mechanisms, such as missing completely at random, missing at random, and missing not at random. Incorporating these distinctions into modeling frameworks remains an important open problem for future research.

#### Applications: Address real unmet medical needs

Most of the techniques considered here have concentrated on metrics such as accuracy and mean squared error when it comes to performance evaluation. We think we need to meet other practical needs in medical applications.

##### Interpretability

In practical clinical applications, models are expected not only to provide predictions (“what”) but also to offer explanations (“why”). Without interpretability, even highly accurate models may be difficult for medical practitioners to trust [120]. Doctors can not treat patients based only on a conclusion without any explanation. Nowadays, most of the DL methods are troubled by poor interpretability [121]. Recent studies have made notable progress in improving the applicability of DL models in medical settings. For instance, attention-based mechanisms [122] and causal analysis approaches [123] provide interpretable insights into model predictions.

##### Reliability

Although many DL models output class probabilities, they often lack proper calibration and do not report metrics such as expected calibration error in classification tasks. In addition, most imputation models do not produce probabilistic outputs. As a result, deterministic models struggle to properly quantify and propagate uncertainty, limiting their reliability in clinical applications [124].

##### Adaptability

Data imbalance is a pervasive issue in medical datasets, where normal cases typically dominate and pathological cases are underrepresented [119]. Models that do not account for this imbalance tend to perform poorly on minority classes. While techniques such as data augmentation [125] have been proposed, synthetic data may not fully capture real-world distributions. Furthermore, limited sample sizes—often due to privacy constraints—pose additional challenges. In this context, approaches such as few-shot learning [126], transfer learning [127], and federated learning [128] represent promising directions for ISMTS modeling.

##### Continuous prediction

Continuous monitoring is critical for managing high-risk patients, enabling timely intervention and better resource allocation. Compared to single-shot predictions, continuous prediction paradigms—such as ongoing diagnosis, prognosis [129], and vital sign control [130]—are more aligned with clinical needs. However, current DL models often suffer from issues such as catastrophic forgetting, overfitting, and delayed responses in continuous prediction settings [131].

## Conclusion

In this review, we provide a comprehensive overview of ISMTS. We first characterize the unique properties of ISMTS in clinical settings and then systematically examine existing modeling approaches from both technology-driven and task-driven perspectives. Within each category, we further organize methods into finer subgroups and summarize their key design principles. In addition, we compare representative approaches through imputation and prediction tasks, highlighting their respective strengths and limitations. Finally, we discuss the key challenges and emerging opportunities in modeling ISMTS data.

## Data Availability

All original data used in this paper are publicly available and can be accessed as follows: MIMIC-III [29], CINC-2012 [89], CINC-2019 [33], and COVID-19 [34]. The code and preprocessed data are publicly available at https://github.com/SCXsunchenxi/ISMTS-Review. Correspondence and requests for materials should be addressed to C.S., S.H., and H.L.
